# The Role of Adjuvant Nonavalent HPV Vaccination After LLETZ in Patients with Isolated CIN2: A Controlled Cohort Study

**DOI:** 10.3390/pathogens15080814

**Published:** 2026-08-01

**Authors:** Vincenzo Pinto, Miriam Dellino, Marco Cerbone, Edoardo Di Naro, Achiropita Lepera, Silvio Tafuri, Gerardo Cazzato, Ettore Cicinelli

**Affiliations:** 1 Department of Interdisciplinary Medicine (DIM), Obstetrics and Gynecology Unit, University of Bari “Aldo Moro”, Piazza Giulio Cesare 11, 70124 Bari, Italy; 2 Department of Interdisciplinary Medicine (DIM), Public Health Unit, University of Bari “Aldo Moro”, 70124 Bari, Italy; 3 Department of Precision and Regenerative Medicine and Ionian Area (DiMePre-J), Pathology Unit, University of Bari “Aldo Moro”, 70124 Bari, Italy

**Keywords:** HPV, human papillomavirus, vaccine, LLETZ, LEEP, cervical conization CIN2, Gardasil vaccine

## Abstract

Background: Women treated for cervical intraepithelial neoplasia grade 2 (CIN2) face an increased risk of disease recurrence due to persistent or newly acquired human papillomavirus (HPV) infections, suggesting a potential role for adjuvant HPV vaccination. While a growing body of literature evaluates the outcomes of HPV vaccination following a large loop excision of the transformation zone (LLETZ) for high-grade squamous intraepithelial lesions (CIN2–3), data specifically focused on isolated CIN2 remain limited. This study aims to evaluate the clinical efficacy of the nonavalent HPV vaccine (9vHPV) as an adjuvant strategy to reduce CIN2+ recurrence in women undergoing LLETZ for histologically confirmed, isolated CIN2. Methods: Between November 2017 and November 2024, women undergoing LLETZ for histologically confirmed CIN2 received their first dose of adjuvant 9vHPV within one month of surgery. Patients who achieved double-negative co-testing (Pap test and high-risk HPV DNA test) at the 6-month post-LLETZ follow-up were included in the study group. Conversely, patients with at least one positive test result at 6 months were excluded to rule out residual disease. The study group underwent a subsequent Pap smear at 12 months and a full co-test at 18 months post-surgery; upon achieving negative results through the 18th month, patients returned to the organized screening program. The primary outcome was the recurrence rate, defined as histologically confirmed CIN2+ occurring more than 6 months post-treatment. It was compared against an unvaccinated historical control group treated with LLETZ prior to the study period. The secondary outcome evaluated the prevalence of HPV genotypes at baseline and at the time of recurrence. Results: In the vaccinated group, 3 women (2.03%) experienced CIN2+ recurrence compared to 6 women (5.71%) in the unvaccinated control group. This represents an absolute risk difference of 3.69% and a relative risk reduction of 64.6%, though the difference did not reach statistical significance via Fisher’s exact test (*p* = 0.165). In the study group, two recurrences were detected at 18 months (associated cytologies: ASC-H and LSIL; genotypes: HPV 51/53 and 16, respectively) and one at four years (cytology: HSIL; genotype: HPV 33/58). In the control group, recurrences occurred at 12 months (*n* = 3), 18 months (*n* = 2), and four years (*n* = 1). Associated cytology revealed HSIL in three cases, ASC-H in one case, LSIL in one case, and ASCUS in the final case. The corresponding HPV genotypes were 16 (two cases), 18, 51, 31 and 56 (co-infection), and one case of high-risk HPV, not further genotyped. Conclusions: Adjuvant 9vHPV administration after LLETZ for isolated CIN2 was associated with a clinically substantial lower absolute recurrence rate (2.03% vs. 5.71%, corresponding to a 64.6% relative risk reduction). Although this reduction did not achieve statistical significance (p=0.165) due to our small sample size and a low baseline recurrence rate in this isolated cohort, the effect size is highly comparable to broader CIN2+ studies. The study was likely underpowered to prove statistical significance, and larger multi-center studies are required to confirm the statistical value of adjuvant vaccination specifically in isolated CIN2 lesions.

## 1. Introduction

The introduction of Human Papillomavirus (HPV) vaccination changed irreversibly the story of cervical cancer (CC), contributing to the shifting of this neoplasia into the field of preventable diseases [1].

In 2020, the World Health Organization (WHO) launched the “90-70-90” global initiative to eliminate (CC) as a public health problem by 2030. This strategy relies on three main pillars: prophylactic vaccination, early diagnosis through high-performance screening, and the timely treatment of cervical precancerous lesions and invasive disease (WHO, 2020). However, disparities in global healthcare access and infrastructure are significantly slowing down progress toward reaching these WHO targets, particularly in low- and middle-income countries. In recent decades, numerous therapeutic advances have been achieved in minimally invasive treatment of high-grade cervical intraepithelial neoplasia (CIN2/CIN3), which are considered immediate precursors to invasive cervical cancer (CC) [2]. Traditional, broad cold-knife conization has largely been replaced by laser conization and, most notably, large loop excision of the transformation zone (LLETZ). As a globally adopted technique, LLETZ allows for a tailored excision performed entirely in an outpatient setting under local anesthesia. Crucially, this approach demonstrates a minimal impact on the long-term risk of subsequent preterm delivery [3]. Although treatment of CIN2-3 by LLETZ is a cost-effective strategy to prevent precancerous lesion’s progression to CC, women undergone excisional therapy for CIN2-3 may harbor persistent HPV infection after treatment and remain susceptible to new HPV infections [4,5]. Although the vast majority of recurrences are detected within the first two years following treatment [6] these women continue to face a significantly elevated long-term risk of cervical cancer for decades [7] underscoring the critical need for effective adjuvant preventive strategies [5]. According to a systematic review and meta-analysis, following LLETZ for CIN2-3 there is an absence of high-grade histological recurrence in 90% of cases at one year [8]. In patients treated for CIN3, the overall risk of recurrence (histological HSIL) ranges from 5% to 17% and the relative risk of developing invasive cancer is 2–5 [8].

The use of different HPV vaccinations (bivalent, tetravalent, nonavalent) as adjuvant strategy following excisional treatment has been evaluated in highly heterogeneous studies. In women who remain susceptible to persistent or newly acquired HPV infection after treatment, vaccines induce a long-lasting protection against involved and new genotypes. Indeed, it is known that HPV-positive women exhibit a high susceptibility to the virus and are prone to rapid reinfection [9]. Remains an open question whether vaccines really boost the effect of excisional treatment [9].

Several observational studies and post hoc analyses have reported a reduction in CIN2+ recurrence after LLETZ among vaccinated women; however, results remain somewhat conflicting and are affected by substantial methodological heterogeneity, partly reflecting the absence of shared, high-quality, evidence-based recommendations. In fact, a survey by the European Federation for Colposcopy investigating the notable inconsistencies in how European nations handle post-treatment care observed marked heterogeneity in clinical practice across Europe regarding adjuvant HPV vaccination. This includes significant disparities in clinical indications, intervention timing, target patient populations, vaccine valency, and national reimbursement policies [10].

CIN2, represents an intermediate histopathological category between CIN1 and CIN3. It is characterized by immature basal-type cells involving the lower two-thirds of the squamous epithelium without stromal invasion [11]. Although the diagnostic criteria for CIN2 are well-defined, its diagnosis is associated with substantial interobserver variability in cervical specimens [12]. This diagnostic uncertainty carries a relevant risk of misclassification and, consequently, inappropriate treatment [13].

CIN2 carries a higher likelihood of spontaneous regression compared with CIN3. According to a metanalysis of Loopik et al. on 42 studies on women affected by CIN2, the overall regression rate was 55% (95% CI = 50–60, I^2^ = 85%). The persistence, and progression rates were 23% (95% CI = 19–28, I^2^ = 83%), and 19% (95% CI = 15–23, I^2^ = 88%), respectively [14].

Evidence in the literature identifies post-treatment vaccination as an effective adjuvant measure in women who have undergone LLETZ. While the exact biological mechanisms driving this protective effect are not yet fully understood, available clinical data consistently demonstrate a reduction in CIN2+ recurrence of greater than 60% among treated and vaccinated individuals compared to those who undergo excisional treatment alone.

A systematic review of 11 studies from Di Donato et al. established that the recurrence of CIN2+ after treatment was significantly lower in the vaccinated compared with the unvaccinated group (OR 0.35; 95% CI 0.21–0.56; *p* < 0.0001) [15]. On the other hand, the reduction rate of HPV persistence observed in the vaccinated compared with the unvaccinated cohorts was not significant (OR was 0.84; 95% CI 0.61–1.15; *p* = 0.28) [15].

According with a 2025 Cochrane’ review, evidence from two randomized controlled trials show that HPV vaccination compared to no HPV vaccination in women undergone LLETZ may reduce the risk of CIN2+ (risk ratio 0.40, 95% confidence interval 0.26 to 0.63 [16]. Overall, the existing evidence for HPV vaccination in patients undergone LLETZ is largely based on non-randomized studies of interventions with serious or critical risk of bias and low-to very low-certainty evidence [16].

A more recent meta-analysis compiled data from 17 distinct studies to evaluate the impact of HPV vaccination following LLETZ [17]. The analysis encompassed a total population of 33,181 patients including a vaccinated cohort of 6665 women and a control cohort of 26,516 unvaccinated women. The pooled data consisted of four randomized controlled trials, eight prospective cohort studies, and five retrospective cohort studies. Metanalysis included studies with bivalent, quadrivalent and nonavalent vaccines. The overwhelming majority of the analyzed literature (12 out of 17 studies) indicates that administering the HPV vaccine before or after surgical excision significantly lowers the risk of high-grade cervical lesion recurrence [17]. In details, meta-analysis, utilizing a random-effects model, demonstrated that adjuvant vaccination was associated with a 62% relative risk reduction for recurrent CIN2+ (RR 0.38, 95% CI 0.29–0.51, *p* < 0.0001). However, a minority of trials, including certain well-controlled randomized studies, observed no statistically significant preventative benefit. Sand et al. reported a non-significant adjusted hazard ratio of 0.86 (95% CI: 0.67–1.09) when comparing vaccinated and unvaccinated cohorts post-conization [18]. Hildesheim et al. found no notable decrease in post-surgical infections or cervical lesions. The calculated vaccine efficacy against post-treatment CIN2+ lacked statistical significance and hovered near zero or negative values [19]. VACCIN Trial [20] is a phase 4, randomized, placebo-controlled trial. It failed to show a statistically significant advantage for post-LEEP 9vHPV vaccination. At 24 months, CIN2–3 recurred in 6% of the vaccine group versus 9% of the placebo group (RR: 0.67, 95% CI: 0.40–1.11) [20].

All studies supporting adjuvant HPV vaccination after excisional treatment included mixed populations of CIN2 and CIN3 lesions, despite substantial biological differences between these entities. If CIN3 is widely recognized as a true precursor of CC with a high risk of recurrence, CIN2 encompasses lesions with heterogeneous malignant potential and higher rates of spontaneous regression. Consequently, pooling CIN2 and CIN3 in clinical trials can indeed mask the true efficacy of an intervention for each distinct group, potentially overestimating the clinical benefit of adjuvant HPV vaccination for women who have isolated CIN2.

Due to the absence of studies focused exclusively on patients who have undergone LLETZ specifically for CIN2, and bearing in mind the natural history of these intraepithelial lesions, we conducted a study aimed at assessing disease recurrence ≥CIN2 in patients treated with LLETZ for CIN2 who were subsequently vaccinated with the 9vHPV vaccine. These data were compared with a control cohort consisting of women who underwent LLETZ but did not receive post-treatment vaccination. The results of this comparative analysis are the subject of the present paper.

## 2. Materials and Methods

A comparative cohort study with historical controls was conducted at the Unit of Obstetrics and Gynecology, University Medical School of Bari. Between November 2017 and November 2024, 211 women underwent large loop excision of the transformation zone (LLETZ) resulting in a histological diagnosis of CIN2.

All patients had previously been diagnosed with CIN via targeted biopsy under colposcopic guidance. In 179 patients (84.83%), the biopsy histology was CIN2. In the remaining 32 cases (15.17%), the biopsy initially indicated CIN1; however, LLETZ was performed because the internal margins of the lesion were not visible (Type 3 Transformation Zone). The subsequent excision histology in these cases revealed CIN2. The interval between biopsy and LLETZ did not exceed two months. All excisional procedures were performed by a single operator (V.P.) using a semicircular electrosurgical loop with a diameter of 2.5–3 cm and a wire thickness of 0.3 mm. The electrosurgical unit was operated in pure cut mode with a power output of approximately 50 W. The cervical specimen was removed as a single fragment in 193 (91.46%) of cases; in the remaining 18 (8.54%), a “top hat” technique was employed, resulting in a two-fragment excision. After loop excision, electrosurgical coagulation of the wound bed was performed selectively only in the presence of hemorrhagic foci, rather than systematically. Laser vaporization was not applied to the crater in any cases.

Adjuvant 9vHPV vaccination Gardasil 9™ (Merck & Co., Rahway, NJ, USA) was administered to all patients at the hospital’s vaccination center following a 0–2–6 month schedule. The first dose was administered within 30 days of surgical treatment. Vaccinations were performed under a full-subsidy program.

All patients were evaluated six months post-treatment via co-testing (Pap test and HR-HPV DNA test). Our unit serves as a tertiary referral center for suspected high-risk cervical lesions, receiving patients from a wide catchment area. Therefore, follow-up HPV testing was performed using three distinct types of HPV assays: the Seegene Allplex™ HPV28 Detection assay (Seegene, Seoul, Republic of Korea), the BD Onclarity™ HPV Assay (Becton, Dickinson & Co., Sparks, MD, USA), and the Roche cobas^®^ 4800 HPV test (Roche Diagnostics, Indianapolis, IN, USA). Additional information about these tests can be found in the Supplementary Materials (Section S1). Patients in the study group who tested negative during the 6-month co-testing underwent a follow-up Pap smear 12 months post-surgery. If results remained negative, a subsequent co-test was performed 18 months after the initial treatment. Upon achieving negative results through the 18th month, patients returned to the organized screening program. In our protocol, this transition involved a follow-up at 3 years using a primary HR-HPV test—rather than the standard 5-year interval—to account for the elevated baseline risk of recurrence.

In cases of positive findings (cytology ASC-US+ and/or a positive HR-HPV test), patients were referred for colposcopy. Any suspicious lesions identified during colposcopic evaluation underwent biopsy, with recurrence defined as a histologically confirmed diagnosis of CIN2+. HPV positivity or abnormal cytology alone were not considered recurrence endpoints.

The incidence of CIN2+ recurrence was subsequently assessed. Recurrence was therefore defined as histologically confirmed CIN2+ occurring at least six months after the initial excisional treatment.

Inclusion criteria were a histological diagnosis of CIN2 on the excisional specimen following LLETZ, no previous HPV vaccination, and eligibility for HPV vaccination.

The results were compared with data retrieved from a retrospective historical control group of 105 unvaccinated women with a histological diagnosis of CIN2, all of whom were treated until October 2017 by the same operator using an identical excisional technique. The control group also included 6 women treated after October 2017 who were not vaccinated due to medical contraindications or refusal for personal beliefs. Patients of the control group underwent the same follow-up protocol of the study group.

The following exclusion criteria were applied to both study groups: persistence of dysplasia at the first evaluation within six months of treatment, association with glandular lesions, HIV infection, use of immunosuppressive therapy, pregnancy occurring within the first two years of follow-up, prior treatment for CIN, HPV vaccination (any type) prior to the study period. For the study group only, a non-compliant vaccination schedule was considered an exclusion criterion.

For both groups, we reviewed the medical chart of the outpatient clinic and confirmed the collected information using a person-to-person phone survey for each patient.

Differences in recurrence rates occurring more than 6 months after treatment between vaccinated and unvaccinated groups were analyzed using Fisher’s exact test; *p* value < 0.05 was considered statistically significant. Microsoft Excel™ for Microsoft 365 MSO (Version 2602) and the Python 3™ software (Python Software Foundation™), along with the lifelines library, were used as the statistical software. A secondary study outcome was the evaluation of HPV genotypes responsible for CIN2, with a specific focus on those identified in women from both groups who experienced CIN2+ recurrence.

## 3. Results

During the period under review, 211 women treated for CIN2 and undergoing adjuvant vaccination were recruited. Four patients were excluded due to positive co-testing at the 6-month follow-up. One patient was excluded due to the association of a glandular lesion (AIS) with the squamous lesion. Six women refused vaccination and were assigned in the control group. Further exclusions included: 17 cases where the first dose of the 9vHPV vaccine was administered more than 30 days after LLETZ, 12 women for failure to comply with the vaccination schedule and three women due to medical conditions (one HIV-positive case and two receiving concomitant immunosuppressive therapy). Consequently, 168 women were eligible for evaluation, of whom 148 were observed for more than 6 months. The composition of the vaccination group and the specific exclusion criteria are summarized in Table 1.

At the 6-month co-testing follow-up, 135 (91.22%) patients had completed the three-dose vaccination schedule, while the remaining 13 (8.78%) had received at least two doses.

The average age of the 148 women in the vaccination group at the time of treatment was 41 years (range 27–64 years). Regarding parity, 70 (47.3%) were nulliparous, 37 (25%) had one child, and 41 (27.7%) had two or more children.

The median cone height at LLETZ was 10.9 mm (range 6–20 mm). In cases of top-hat excision, the cumulative cone height was calculated. Surgical margins were free of disease in 83% of patients. Follow-up durations extended from 1 to 8.5 years.

In this study group, three histologically documented recurrences were observed, accounting for 2.03% of the 148 patients followed for more than six months. Two recurrences were detected at the 18-month co-testing follow-up, while the third was identified after four years, following the patient’s transition to a local screening program. Cytological analysis at the time of recurrence revealed ASC-H in the first case, HSIL in the second, and LSIL in the third. The associated HPV genotypes were 51 and 53, 33 and 58, 16 respectively (Table 2).

The control group consists of 143 women who underwent LLETZ with a definitive histological diagnosis of CIN2, but who were not vaccinated because they were treated until October 2017. After applying the same exclusion criteria used for the study group, 105 patients remained eligible for follow-up and comparison with the vaccinated cohort. The average age of control group at the time of treatment was 36 years (range 28–61 years). Regarding parity, 44 (41.9%) were nulliparous, 29 (27.61%) had one child, and 32 (30.47%) had 2 or more children. The median cone height at LLETZ was 11.3 mm (range 7–23 mm). Surgical margins were free of disease in 85% of patients. Evaluation of p16 expression via immunohistochemistry was not considered because it was performed inconsistently and only in a minority of patients. The follow-up duration was extended from 2 years to 19 years (this group included 6 women treated from November 2017 who were not vaccinated).

In this group, six histologically documented recurrences were observed, representing 5.71% of cases. Three recurrences were detected at the 12-month follow-up, two at the 18-month follow-up, and one four years after LLETZ during the local screening program. Associated cytology revealed HSIL in three cases, ASC-H in one case, LSIL in one case, and ASC-US in the final case. The corresponding HPV genotypes were 16 (two cases), 18, 51, 31 and 56 (co-infection), and one case of high-risk HPV not further genotyped (Table 2).

**Figure 1:**
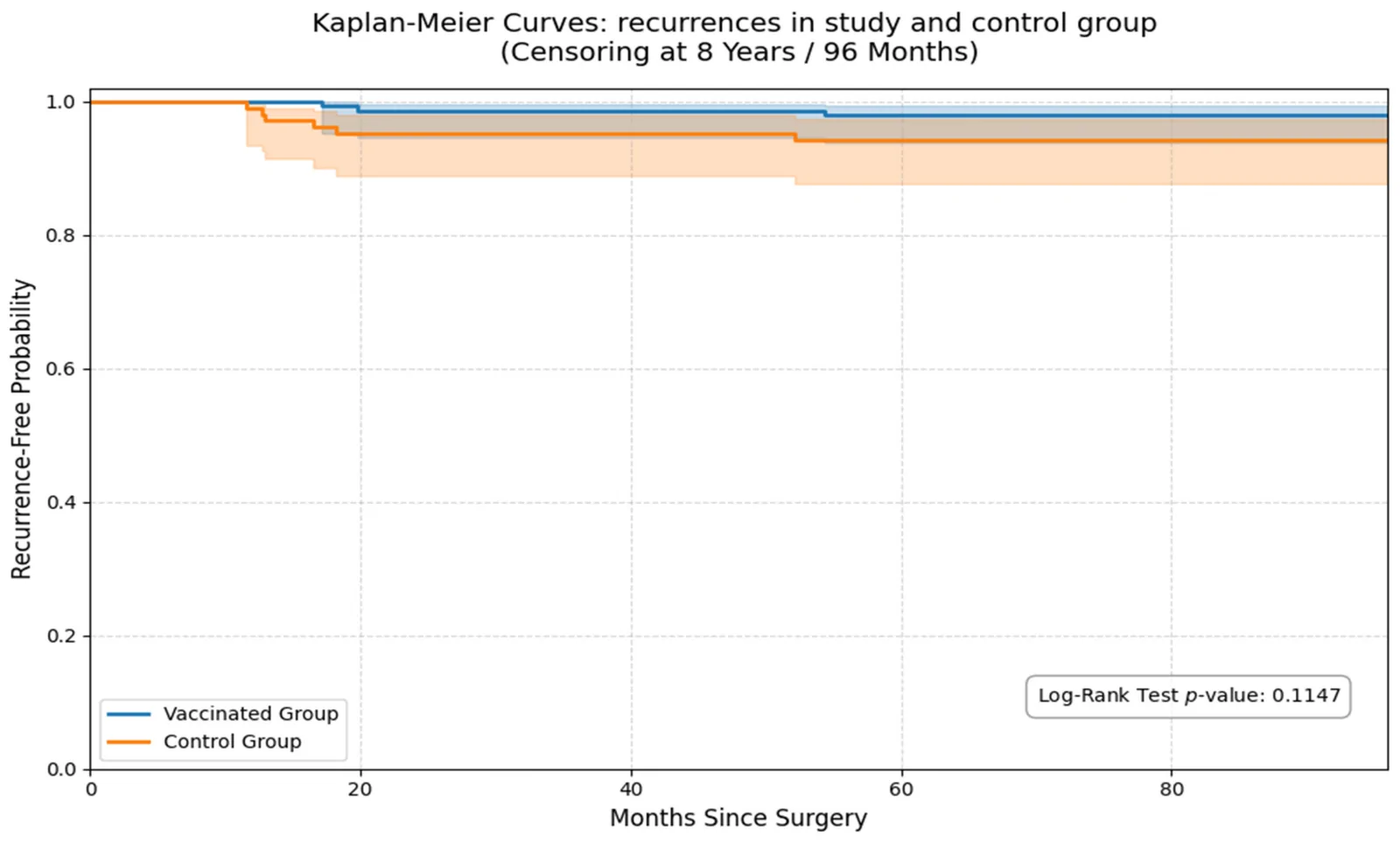
Kaplan–Meier estimates of recurrence-free survival (RFS) comparing the study group and control group over a 96-month period. Shaded areas represent the 95% confidence intervals. Administrative censoring was strictly applied at 8 years (96 months). The Log-Rank test indicates no statistically significant difference between the two cohorts (p=0.1147).

The results indicate a recurrence rate of 2.03% in the group of women who underwent LLETZ followed by adjuvant 9vHPV vaccination, compared to 5.71% in women treated with LLETZ alone prior to 2017. This represents a 64.6% reduction in the risk of recurrence for the vaccinated group (reduction = not vaccinated rate—vaccinated rate/not vaccinated rate × 100). The difference did not reach statistical significance according to Fisher’s Exact Test (*p* = 0.165). The clinical impact was further evaluated using the Number Needed to Treat (NNT). The Absolute Risk Reduction (ARR) was 3.69% (5.71–2.02%), resulting in an NNT of 27. (Table 3). To address potential bias from differing follow-up durations between the cohorts, we also employed a time-to-event framework to ensure a rigorous, unbiased comparison. A Kaplan–Meier survival analysis was performed to evaluate recurrence-free survival (RFS) over an 8-year (96-month) follow-up period. The cumulative RFS curves for both the study and control groups followed an identical trajectory during the initial 20 months post-intervention. Beyond this point, the curves began to diverge, with the study group exhibiting a higher proportion of patients remaining free from disease recurrence compared to the control group. (Figure 1). While this points toward a positive clinical trend, the difference in RFS between the two cohorts did not reach statistical significance via the Log-Rank test (*p* = 0.1147). This outcome is consistent with the visible overlap of the 95% confidence intervals throughout the follow-up period, suggesting that while a clinical trend is visible, a larger sample size may be required to definitively confirm a statistically significant advantage.

**Figure 2:**
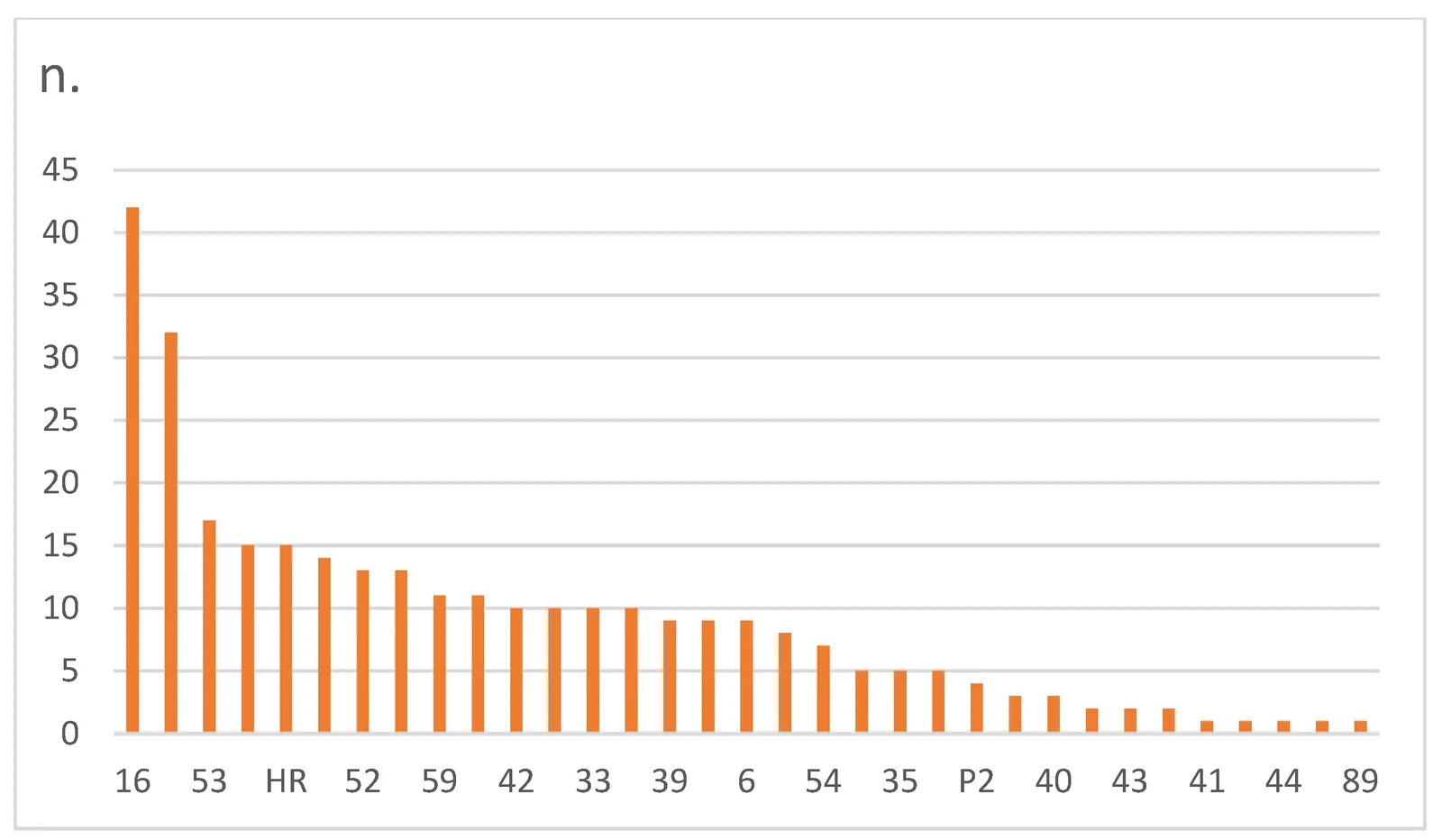
Distribution of HPV genotypes in the population of treated and vaccinated women. HR: high-risk HPV genotypes 16, 18, 31, 33, 35, 39, 45, 51, 52, 56, 58, 59, 66, 68, group P1: 33, 58, group P2: 56, 59, 66, group P3: 35, 39, 68.

The control group consists of 105 women and includes 6 women treated after October 2017 who refused vaccination or had contraindications. The timeline overlap of the cohort distribution was handled statistically by performing a sensitivity analysis, excluding the 6 contemporary controls and comparing the outcomes of the vaccinated group solely against the 99 strictly historical controls. Because zero recurrences occurred among the 6 contemporary controls, their exclusion does not alter our clinical findings or statistical conclusions. Primary analysis on 105 cases (99 historical + 6 contemporary) detected 6 recurrences (recurrence rate 5.71%), with a relative risk reduction of 64.6%. Sensitivity analysis on 99 strictly historical controls detected six recurrences (recurrence rate 6.06%), with a relative risk reduction of 66.5%. Therefore, excluding the contemporary control cohort yields highly similar recurrence rates (6.06% vs. 5.71%) and relative risk reductions (66.5% vs. 64.6%), with no change in the overall statistical significance. The timeline overlap did not introduce bias or alter the study’s primary conclusions. As a secondary outcome of the study, genotype analysis was conducted to evaluate the prevalence of associated HPV strains in the vaccinated group. The most frequently detected genotypes were HPV 16 and 31, respectively detected in 42 and 32 out of 148 women. In 15 women specific high-risk genotyping was not performed; however, the samples tested positive for a high-risk pool comprising genotypes 16, 18, 31, 33, 35, 39, 45, 51, 52, 56, 58, 59, 66 and 68. (Table 4, Figure 2).

## 4. Discussion

Traditionally, clinical guidelines across most nations favor surgical removal for CIN2 to prevent potential malignant transformation although excisional procedures carry a documented risk of subsequent preterm labor, and spontaneous regression of CIN2 is frequently observed—particularly in younger populations. After 2 years, the regression rate of untreated CIN2 is up to 55% [13,14,21]. Consequently, many healthcare systems now consider active surveillance as a viable alternative for younger patients. While monitoring rather than operating mitigates the issues surrounding clinical overtreatment, it introduces other challenges; undergoing active surveillance has been shown to induce psychological distress but, crucially, a subset of patients will inevitably progress to CIN3 lesions. A large retrospective Danish study comprising 4585 women (<40 years) not surgically treated -of whom 12.7% were vaccinated within 6 months after the diagnosis of CIN2- demonstrated a progression to CIN3+ in 30.3% of all cases. No protective effect was proven within the vaccinated subgroup [22]. Results from the same study group demonstrated that receiving at least one dose of an HPV vaccine (whether bivalent, quadrivalent, or nonavalent) was associated with a statistically significant decrease in the risk of disease progression among women with untreated CIN2 only when administered before age 20 [23].

Treatment with LLETZ is considered the primary therapeutic strategy for patients with CIN2 (Perkins), particularly for patients over 30 or when the squamocolumnar junction is not fully visible. The overall risk of recurrence after LLETZ treatment is hard to define. In fact, CIN2 and CIN3 are grouped together under the umbrella of “high-grade squamous intraepithelial lesions” (HSIL) because clinical management protocols treat them identically, although the natural story and the risk of invasion is very different. Cumulative data of recurrences for CIN2-3 ranges between 4% and 8% in large cohort studies, with some broader literature citing a 2% to 17% range depending on the population and follow-up length [6,24]. The vast majority of recurrences are detected within the first 2 years post-procedure.

Data considering the recurrence of isolated CIN2 after LLETZ is available in the medical literature, though it is extracted from broader CIN2-3 lesion cohorts. Studies isolating patients who had a baseline diagnosis of isolated CIN2 report recurrence/persistence rates generally ranging between 2.5% and 5%. A large retrospective study by Ding et al., which analyzed over 4300 patients, found that the post-LLETZ recurrence risk was 3.8% in CIN2 and 8.2% in CIN3 patients. Using isolated CIN2 as the baseline reference group, this difference corresponds to a significantly higher risk of recurrence for CIN3 patients, with a hazard ratio (HR) of 1.45 [25].

It is even more challenging to assess the risk of disease recurrence in women who undergo adjuvant vaccination after LLETZ for isolated CIN2. All currently available evidence derives from heterogeneous cohorts of women with CIN2–3, without specific stratification or analyses dedicated to those with isolated CIN2. Furthermore, existing systematic reviews and many studies often pool results across different excisional techniques, variable or unspecified vaccination timelines, and bivalent or quadrivalent vaccines. To date, no study has been specifically designed to evaluate the effectiveness of adjuvant HPV vaccination exclusively in women with isolated CIN2, thereby limiting the applicability of current data to this distinct subgroup. To the best of our knowledge, this is the first study investigating the recurrence risk of isolated CIN2 lesions, treated with LLETZ and vaccinated with 9vHPV within one month of surgery.

We conducted a retrospective, single-center, comparative cohort study evaluating our experience over the last eight years. The study population consisted of two groups: a retrospective cohort of women who received the 9vHPV vaccine post-LLETZ, and a retrospective historical control group of unvaccinated women treated during the same period. This design allowed for a comparison of recurrence rates while maintaining the current clinical standard of care.

Since all women referred for LLETZ had undergone a prior biopsy no more than two months earlier, we faced a dilemma during the study design: whether to consider the CIN2 finding at the time of biopsy or at the time of LLETZ.

Using the biopsy findings has the advantage of including cases where a small extrajunctional lesion is completely removed by the biopsy itself. In fact, some women with biopsy-confirmed CIN2 showed only CIN1 or were even negative for dysplasia upon LLETZ excision. Following this method also allows for vaccination within a shorter timeframe.

Conversely, considering only cases where CIN2 is confirmed via LLETZ allows for the inclusion of patients whose biopsy indicates CIN1, but who undergo LLETZ due to a Type 3 Transformation Zone which subsequently reveals CIN2. This approach also permits the analysis of margin involvement as a variable in disease recurrence. However, this method results in a delay in accessing vaccination.

Including both groups would have created methodological issues in the interpretation of the results. Since prioritizing the post-LLETZ diagnosis results in fewer lost cases, we adopted this method, excluding from the study those cases with biopsy-confirmed CIN2 that were not confirmed following LLETZ. However, for the sake of consistency in our evaluation, we also included 6 eligible patients, 4 in the vaccination group and 2 in the control group) who were initially diagnosed with CIN3 on biopsy but were ultimately diagnosed as CIN2 after LLETZ.

This study does not aim to establish the absolute recurrence rate of CIN2 following LLETZ; rather, it focuses specifically on the clinical benefits of HPV vaccination in women treated for this condition. Notably, cases of disease persistence within six months of surgery were excluded from the analysis. Given that the vaccination cycle is completed over approximately six months, and although a robust and consistent antibody response is detectable much earlier [1], we chose to consider only recurrences that appeared after this time frame to ensure the evaluation of the vaccine’s prophylactic effect. By excluding patients with positive 6-month co-testing, we aimed to focus specifically on the long-term protective effect of adjuvant vaccination in patients who initially achieved clinical clearance. HPV positivity or cytological abnormalities alone were not considered recurrence endpoints, in order to avoid overestimation of clinically relevant disease, particularly in our cohort of women with CIN2, in whom transient HPV persistence is common. This approach allowed evaluation of clinically meaningful outcomes rather than virological or cytological persistence.

Since we excluded women with a positive co-test at the 6-month follow-up we recognize that the absolute recurrence rate of CIN2 following LLETZ in our cohorts may be underestimated. This exclusion was intentional to distinguish true disease recurrence from persistence related to incomplete primary excision.

The results show a recurrence rate of 2.03% in the group of women who underwent LLETZ and vaccination, compared to 5.71% in women treated with LLETZ alone. This corresponds to a 64.6% reduction in the risk of recurrence for the vaccinated group. While this represents a nearly three-fold reduction in the clinical incidence of recurrence, the difference did not reach statistical significance according to Fisher’s Exact Test (*p* = 0.165). The lack of a significant *p*-value is likely due to the low absolute number of events (n = 9 total recurrences across both groups), which limits the statistical power of the analysis. However, despite the lack of statistical significance, the clinical impact remains notable; the absolute risk reduction (ARR) was 3.69%, yielding a number needed to treat (NNT) of 27. This indicates that for every 27 women undergoing LLETZ for CIN2, one histologically documented recurrence can be prevented by administering the adjuvant 9vHPV vaccine. The trend clearly favors adjuvant vaccination and aligns with larger multi-center studies that have demonstrated the protective effect of vaccination post-LLETZ in pooled high-risk lesions.

Only few studies evaluated the 9vHPV vaccination following excisional treatment for CIN2-3. Henere et al. in a prospective study observed a significant lower HSIL recurrence when vaccination occurred before LLETZ (0.9% vs. 6.5%; *p* = 0.047). However, has to be considered that the mean time of the follow-up to the last control was quite limited (20.2 months; SD 10.6) [26]. Dvořák et al., in a retrospective, monocentric cohort study, reported a 90% reduction in CIN2+ recurrences (95% CI: 12–99%) among 9vHPV-vaccinated participants compared to women undergoing surgical excision alone. Additionally, a 42% risk reduction was noted for patients with positive surgical margins, although this subgroup analysis did not reach statistical significance (*p* = 0.661) [27]. A study by Anton et al. evaluated women who underwent surgical excision resulting in histologically negative margins and who subsequently received the 9vHPV vaccine [Anton]. Although viral clearance was achieved by all women vaccinated prior to conization and by 94.9% of those vaccinated at the time of surgery, the study reported an appreciable, though not statistically significant, 42% reduction in CIN2+ recurrence rates within the vaccinated group [28]. On the contrary, Van de Laar et al., in a multicenter, double-blind, placebo-controlled, randomized phase 4 trial in the Netherlands (the VACCIN study), failed to demonstrate a statistically significant advantage for adjuvant 9vHPV vaccination. Over a 24-month follow-up period, CIN2–3 recurred in 6% of the vaccine group compared to 9% of the placebo group (RR: 0.67, 95% CI: 0.40–1.11) [20].

In the systematic review from Maiorano et al. these three studies [20,26,27] with the 9vHPV vaccine (n = 757 vs. 597) yielded a pooled RR of 0.41 (95% CI 0.18 –0.95) [17].

When considering the timing of recurrences, in the control group they appeared earlier, with 50% of cases detected by the 12-month follow-up, compared to the vaccination group where no recurrences were observed prior to 18 months. In the study group, recurrences were associated with both vaccine-targeted and non-vaccine-targeted HPV genotypes. Specifically, two cases involved genotypes included in the nonavalent series (HPV 16, 33, and 58), while one recurrence was associated with HPV 51 and 53, which are not covered by the 9vHPV vaccine. This highlights that while adjuvant vaccination is a potent tool in reducing post-treatment risk, clinical vigilance remains essential as patients remain susceptible to high-risk types not included in current vaccine formulations.

The predominance of HPV 16 among recurrent lesions aligns with historical evidence linking this genotype to persistence and recurrence after treatment [29]. This finding supports the concept of genotype-based risk stratification and suggests that future studies should explore whether adjuvant vaccination may be more effective in selected high-risk subgroups.

The exact mechanism driving the protective effect of the HPV vaccine in patients who have undergone LLETZ is not fully understood, and several hypotheses have been proposed. Before surgery, a high-grade CIN lesion acts as an “immunological blind spot” because the virus effectively suppresses local immune signals [30]. Chronic HPV infection is associated with a modified local microenvironment that prevents the host from mounting a strong antibody response [31]. LLETZ physically cuts out the high viral load and the transformed high-grade CIN cells [32], acting as a crucial biological “reset.” While the surgery removes the localized disease, it exposes raw tissue margins down to the stroma. The antibodies induced by the vaccine subsequently flood the healing cervical tissue and immediately bind to floating, residual viral particles, preventing them from invading newly exposed healthy cells [18]. From an immunological standpoint, the inflammatory response observed in the cervix post-LLETZ is comparable to that seen in patients without active HPV infections rendering the surgical environment an ideal prerequisite for adjuvant vaccine intervention [33].

Many recurrences of high-grade CIN after surgery do not represent a failure of the surgery itself. Instead, they are caused by exposure to new infections from a partner carrying the same or a different high-risk HPV genotype. Also auto-inoculation from residual infections in other lower genital tract migrating back to the healing cervix has to be considered. In both scenarios the adjuvant vaccine creates a persistent baseline of antibodies against HPV capsid protein L1 virus particles that effectively blocks new viral exposures or fresh virus particles produced by remaining infected cells from taking hold in the vulnerable, healing cervical epithelium.

Finally, the adjuvant vaccine may actively boost the host’s immune response. Emerging evidence demonstrates that the vaccine triggers a systemic spike in helper T cells and memory B cells and stimulates cell-mediated immunity, which may play a primary role in the clearance of the viral infection [34].

The strengths of this study lie in several key methodological factors. First, the inclusion criteria were strictly controlled, selecting only patients with isolated CIN2, not previously vaccinated, who received the 9vHPV vaccine within 30 days of LLETZ. Second, the study has an extensive follow-up period, reaching up to eight years in the vaccinated cohort and up to 19 years in the unvaccinated control group. Finally, to eliminate surgical confounding, all LLETZ procedures were performed by a single operator using the identical technique across both groups.

Our study also is subject to some limitations. First, it has a relatively small sample size and may have been underpowered to detect a statistically significant difference in recurrence rates. In details, a key limitation is the risk of a Type II error due to limited statistical power, driven by the low absolute number of recurrence events (n=9). While a clinically notable relative risk reduction of 64.6% was observed in the vaccinated cohort, the difference did not achieve statistical significance. A post hoc sample size calculation, maintaining the current 1.41:1 allocation ratio (148:105 patients), indicates that to detect an absolute risk reduction of 3.69% (assuming a baseline recurrence rate of 5.71% in the control group vs. 2.03% in the study group) with 80% power and a two-sided significance level of 0.05, a total sample size of approximately 837 patients (490 in the study group and 347 in the control group) would be required. However, the clinical effect size (NNT = 27) suggests a strong protective trend, and our findings should be interpreted as hypothesis-generating, highlighting the need for larger, adequately powered multi-center trials to definitively establish the efficacy of adjuvant 9vHPV vaccination in isolated CIN2 cases. Furthermore, using a historical control group might have introduced selection bias, as the two cohorts were managed at different times, even if they both respected the standard of care. Moreover, the cohorts may have been slightly different in their baseline clinical characteristics, such as racial and ethnic composition, lifestyle factors (smoking, diet), or other unknown conditions.

## 5. Conclusions

In women undergoing LLETZ for isolated CIN2, adjuvant 9vHPV vaccination administered after excision was associated with a 64.6% reduction in recurrence risk. This value is highly consistent with the >60% risk reduction reported in broader, larger-scale CIN2+ meta-analyses. Although this reduction failed to achieve statistical significance, it suggests a strong trend toward a protective effect. Nonavalent vaccine appears less effective than in CIN3 because CIN2 exhibits a lower intrinsic potential for progression and, conversely, a higher frequency of resolution following LLETZ alone. Clinically, the low recurrence rate observed in both vaccinated and unvaccinated women confirms that LLETZ alone remains highly effective for isolated CIN2. However, vaccination is a low-burden intervention that may decrease repeat surgeries and contribute to reducing the overall risk of CC. These findings support the clinical recommendation to offer 9vHPV vaccination following LLETZ. While large, adequately powered randomized trials specifically targeting women with isolated CIN2—incorporating HPV genotype stratification and long-term follow-up—are needed to definitively clarify the clinical role of adjuvant vaccination, such studies face significant hurdles. Future prospective randomization will be ethically challenging to propose given the mounting evidence of clinical efficacy in vaccinated cohorts. Furthermore, any future comparison between vaccinated and unvaccinated women will become increasingly difficult due to the progressive, and welcome, shrinking of the unvaccinated population.

## Figures and Tables

**Table 1:** Study Population and Exclusion Criteria Report.

Stage/Exclusion Criteria	Patient Count (n)	Percentage (%)
Total Patients Recruited	211	100.0%
• Delayed first dose (>30 days post-LLETZ)	17	8.1%
• Failure to comply with schedule	12	5.7%
• Vaccination refusal	6	2.8%
• Positive co-testing at 6 months	4	1.9%
• Immunosuppression	2	0.9%
• Associated glandular lesions	1	0.5%
• HIV positivity	1	0.5%
Total Excluded	43	20.4%
Women Eligible for Evaluation	168	79.6%
Women with Follow-up > 6 Months	148	70.1%

**Table 2:** Clinical characteristics of patients (study and control group) with recurrent CIN2+ after 6-month follow-up.

**Vaccinated Group (Study Cohort)**					
**Patient ID**	**Age at LLETZ**	**Cytology and HPV at LLETZ**	**Margins Involved**	**Months at Recurrence**	**Cytology and HPV at Recurrence**
1	42	HSIL, 51/53/70	Endocervical	18	ASC-H, 51/53
2	35	LSIL, HR	No	54	HSIL, 33/58
3	43	ASC-H, 16	No	18	LSIL, 16
**Control Group Cohort**					
**Patient ID**	**Age at LLETZ**	**Cytology and HPV at LLETZ**	**Margins Involved**	**Months at Recurrence**	**Cytology and HPV at Recurrence**
1	29	ASC-US, HR	No	18	LSIL, HR
2	33	HSIL, 18/31/44/52/58	Endocervical	12	HSIL, 18
3	38	ASC-H, 16/51	No	12	HSIL, 16
4	30	ASC-US, 51/66	No	18	ASC-H, 51
5	50	HSIL, 16/35	Endocervical	12	HSIL, 16
6	43	LSIL, HR	No	52	ASC-US, 31/56

**Table 3:** Statistical analysis.

Statistics	Value	95% C.I.
Control Group Recurrence	5.71% (6/105)	2.1–12.0%
Vaccination Group Recurrence	2.03% (3/148)	0.4–5.8%
Fisher’s Exact Test (*p*)	0.165	—
Relative Risk (RR)	0.35	0.09–1.36
NNT (Number Needed to Treat)	27	14–427
Log-Rank test (*p*)	0.1147	—

**Table 4:** Distribution of HPV genotypes in the population of treated and vaccinated women.

HPV Genotype	Number of Observations (n)	Percentage of Total (%)
16	42	28.38
31	32	21.62
53	17	11.49
66	15	10.14
HR	15	10.14
51	14	9.46
52	13	8.78
56	13	8.78
59	11	7.43
73	11	7.43
42	10	6.76
18	10	6.76
33	10	6.76
58	10	6.76
39	9	6.08
82	9	6.08
6	9	6.08
68	8	5.41
54	7	4.73
70	5	3.38
35	5	3.38
P1	5	3.38
P2	4	2.70
45	3	2.03
40	3	2.03
P3	2	1.35
43	2	1.35
11	2	1.35
41	1	0.68
61	1	0.68
44	1	0.68
38	1	0.68
89	1	0.68

HR: high-risk HPV genotypes 16, 18, 31, 33, 35, 39, 45, 51, 52, 56, 58, 59, 66, 68, group P1: 33, 58, group P2: 56, 59, 66, group P3: 35, 39, 68.

## Data Availability

All data in the present study are in the main manuscript.

## Supplementary Materials

The following supporting information can be downloaded at: https://www.mdpi.com/article/10.3390/pathogens15080814/s1, File S1: HPV testing methodology: principle, quality controls, types of HPV detected.

## Abbreviations

The following abbreviations are used in this manuscript:

Abbreviation	Full term
HPV	Human Papillomavirus
9vHPV	Nonavalent Human Papillomavirus Vaccine
CIN2	Cervical Intraepithelial Neoplasia Grade 2
CIN2+	Cervical Intraepithelial Neoplasia Grade 2 or worse
CIN2–3	Cervical Intraepithelial Neoplasia Grades 2–3
LLETZ	Large Loop Excision of the Transformation Zone
CC	Cervical Cancer
WHO	World Health Organization
NILM	Negative for Intraepithelial Lesion or Malignancy
ASC-US	Atypical Squamous Cells of Undetermined Significance
ASC-H	Atypical Squamous Cells—Cannot Exclude High-Grade Squamous Intraepithelial Lesion
LSIL	Low-Grade Squamous Intraepithelial Lesion
HSIL	High-Grade Squamous Intraepithelial Lesion
SCC	Squamous Cell Carcinoma
AGC	Atypical Glandular Cells
AGC-NOS	Atypical Glandular Cells, Not Otherwise Specified
AGC-FN	Atypical Glandular Cells, Favor Neoplasia
AIS	Adenocarcinoma In Situ
ADC	Adenocarcinoma
ECC	Endocervical Curettage
TZ	Transformation Zone
