# Supplementary material for: The Role of Adjuvant Nonavalent HPV Vaccination After LLETZ in Patients with Isolated CIN2: A Controlled Cohort Study
Source: Pathogens. 2026 Aug 1;15(8):814. doi: 10.3390/pathogens15080814 (PMC13516261; doi:10.3390/pathogens15080814)
Supplement: Supplementary file 1 [file pathogens-15-00814-s001.zip › pathogens-4380829-supplementary.pdf]

Supplementary Materials (File S1)

The Seegene Allplex™ HPV28 Detection assay provides full, individualized genotyping for both high-risk (HR) and low-risk (LR) HPV types. It is a multiplex real-time polymerase chain reaction (PCR) test that utilizes advanced MuDT™ (Multiple Detection Temperatures) technology to provide specific cycle threshold values for each of the 28 genotypes within just two reaction tubes. Specifically, the test differentiates 28 distinct HPV strains, classifying them into high-risk oncogenic and low-risk types across two dedicated reaction panels: Panel A comprises the 14 primary HR types (HPV 16, 18, 31, 33, 35, 39, 45, 51, 52, 56, 58, 59, 66, and 68), while Panel B contains 5 probable HR types (HPV 26, 53, 69, 73, and 82) alongside 9 LR genotypes (HPV 6, 11, 40, 42, 43, 44, 54, 61, and 70). The BD Onclarity™ HPV Assay is an automated, qualitative *in vitro* nucleic acid amplification test performed on either the BD Viper™ LT or BD COR™ Systems. Notably, unlike traditional PCR assays that target the *L1* structural gene, it targets the *E6* and *E7* oncogenes. Targeting *E6*/*E7* reduces the potential risk of false-negative results due to viral DNA integration into the host genome—a process that frequently deletes or disrupts the structural *L1* gene. The BD Onclarity™ assay is clinically validated to detect 14 HR-HPV genotypes. Rather than offering a single massive pool, it features a unique "extended genotyping" layout divided into distinct channels: individual genotyping for 6 HR types (HPV 16, 18, 31, 45, 51, and 52), and pooled group genotyping for the remaining 8 HR types divided into 3 risk-stratified groups: Group P1 (HPV 33 and 58), Group P2 (HPV 56, 59, and 66), and Group P3 (HPV 35, 39, and 68). The Roche cobas® 4800 HPV test is an automated, qualitative *in vitro* nucleic acid amplification test. It utilizes automated target enrichment followed by real-time PCR to amplify a sequence of approximately 150 nucleotides within the *L1* region of the HPV genome. The assay employs sequence-specific oligonucleotide probes labeled with distinct fluorescent dyes (TaqMan technology). This allows for the simultaneous amplification and real-time detection of high-risk HPV DNA genotypes in a single analytical run. The assay is clinically validated to detect 14 high-risk HPV genotypes. It explicitly isolates and identifies HPV 16 and HPV 18 via distinct optical channels, while concurrently detecting a pooled aggregate of 12 other high-risk genotypes in a single, separate channel: HPV 31, 33, 35, 39, 45, 51, 52, 56, 58, 59, 66, and 68.
